# The total mass, number, and distribution of immune cells in the human body

**DOI:** 10.1073/pnas.2308511120

**Published:** 2023-10-23

**Authors:** Ron Sender, Yarden Weiss, Yoav Navon, Idan Milo, Nofar Azulay, Leeat Keren, Shai Fuchs, Danny Ben-Zvi, Elad Noor, Ron Milo

**Affiliations:** ^a^Department of Plant and Environmental Sciences, Weizmann Institute of Science, Rehovot 76100, Israel; ^b^Department of Molecular Genetics, Weizmann Institute of Science, Rehovot 76100, Israel; ^c^Department of Molecular Cell Biology, Weizmann Institute of Science, Rehovot 76100, Israel; ^d^Pediatric Endocrine and Diabetes Unit, Edmond and Lily Safra Children’s Hospital, Sheba Medical Center, Ramat Gan 52621, Israel; ^e^Department of Developmental Biology and Cancer Research, Institute for Medical Research Israel-Canada, The Hebrew University-Hadassah Medical School, Jerusalem 91120, Israel

**Keywords:** immune cells, distribution, lymphocyte, macrophage, total mass

## Abstract

The immune system is a complex network of cells with critical functions in health and disease. However, a comprehensive census of the cells comprising the immune system is lacking. Here, we estimated the abundance of the primary immune cell types throughout all tissues in the human body. We conducted a literature survey and integrated data from multiplexed imaging and methylome-based deconvolution. We also considered cellular mass to determine the distribution of immune cells in terms of both number and total mass. Our results indicate that the immune system of a reference 73 kg man consists of 1.8 × 10^12^ cells (95% CI 1.5–2.3 × 10^12^), weighing 1.2 kg (95% CI 0.8–1.9). Lymphocytes constitute 40% of the total number of immune cells and 15% of the mass and are mainly located in the lymph nodes and spleen. Neutrophils account for similar proportions of both the number and total mass of immune cells, with most neutrophils residing in the bone marrow. Macrophages, present in most tissues, account for 10% of immune cells but contribute nearly 50% of the total cellular mass due to their large size. The quantification of immune cells within the human body presented here can serve to understand the immune function better and facilitate quantitative modeling of this vital system.

## Significance

We characterized the human body’s immune cells distribution and provided its total weight. Our findings show that an average individual’s immune system consists of approximately 1.8 trillion cells, weighing around 1.2 kg. Lymphocytes make up 40% of the total number of immune cells and 15% of their mass. Similarly, neutrophils account for comparable proportions. Notably, macrophages constitute 10% of immune cells but contribute nearly 50% of the total cellular mass due to their large size. This knowledge gives an integrative quantitative view of the immune system and facilitates the development of models.

The immune system protects the body from pathogens and foreign substances and comprises a complex network of cells, tissues, and organs. The distribution of immune cells within the body is an essential determinant of immune function and overall health. Different cells have different roles and must be present in suitable numbers to mount an effective response. However, the distribution is challenging to characterize holistically due to the heterogeneity of immune cell populations and the complex organization of immune tissues and organs.

Despite the wealth of studies investigating the human immune system from different angles, there is a need for a comprehensive census of the distribution and mass of the various immune cell types. Previous studies have either focused on specific tissues or cell types (1) or included immune cells in the overall tally (2–4) without detailed resolution.

Previous quantitative studies have relied on various methods, such as flow cytometry and histology. However, these studies have often focused on specific tissues or organs and have used different methods and criteria for quantifying immune cells, making it difficult to compare results across studies. Moreover, analyses were often based on rodents, limiting their generalisability to humans.

One question that has been the subject of previous attention is which organ is the most immunogenic in the human body. Some studies have suggested the gut (5, 6), while others have proposed alternative candidates (7).

In this study, we provide a comprehensive overview of the distribution of immune cells in the human body, aiming to be as rigorous and comprehensive as the available data allow. We explore the tissues and organs where immune cells reside, integrate the available literature, and analyze data from recent sources using descriptive statistics and meta-analysis techniques. We also consider factors influencing immune cell distribution, including age and sex. We aim to provide insights into the immune system’s complex and dynamic organization and shed light on the factors that regulate immune cell distribution in health and disease.

## Materials and Methods

### Types of Immune Cells.

The group of human immune cell types is diverse, and their various categories depend on the resolution of the research. This study focused on the major cell types defined in immunology textbooks (8, 9), divided into two main lineages: lymphoid and myeloid.

We consider four main lymphocyte cell types: T cells, B cells, NK cells, and plasma cells. Although plasma cells are closely related to B cells, they are considered separately in this study to enable a review of previous claims about their distribution (10). The study does not distinguish subpopulations of lymphocytes, such as CD4 or CD8 positive T cells, regulatory T cells, or other innate lymphoid cells besides NK cells.

As for myeloid cells, this study examines four types of granulocytes: neutrophils, eosinophils, basophils, and mast cells. Three nongranulocyte myeloid cell types are also considered: macrophages, monocytes, and dendritic cells. However, the study does not resolve further subpopulations of these cell types, such as classic or nonclassic monocytes or M1/M2 macrophages. Tissue-specific macrophages such as microglia, Kupffer cells, and Langhans cells are considered macrophages without distinction except for estimating cellular mass, which varies among the different tissues.

### Definition of the Reference Person and Variation across the Population.

In our survey, we utilized the standard reference human, historically characterized as a male between 20 and 30 y old, weighing 73 kg, and having a height of 176 cm (11). To qualify our standard reference, we added the term “healthy,” as the individual’s health affects the immune cell population.

Most of the relevant data available in the literature were not stratified by sex and age, and thus, our estimates of the number of cells per gram of tissue mass (henceforth “cell density”) represent a broad normal range independent of sex and age. We use the cell density estimates to extrapolate our results to other segments of the population based on the reference mass of their tissues and organs (11). Thus we obtain a reference estimate for the immune cell population of healthy adult females weighing 60 kg and reference children of age 10 weighing 32 kg (11).

### Estimation of the Immune Cell Population in Various Tissues.

By employing a combination of three primary methodologies, we derived estimates of the population of immune cells present in various tissues throughout the human body.

#### Histology literature–based estimates.

Our goal was to create a database focused on estimating specific tissues’ immune cell density (i.e., number of cells per 1 gram of tissue), primarily using histology and flow cytometry techniques. To achieve this, we conducted a thorough review of existing literature for each immune cell type, aiming to identify studies that had characterized its distribution in different tissues and organs of the human body. Our search was conducted on electronic databases: Google Scholar and PubMed, using a combination of keywords related to the specific immune cell type and relevant tissues and organs. The keywords we utilized included terms such as “population,” “number,” “density,” “histology,” and “flow cytometry.”

Previous estimates for the total number of any specific immune cell were reviewed and transformed into cell density estimates based on the volume or mass of the tissue. In cases in which the primary source of the estimate contained sufficient information, it was used for direct derivation of the cell density instead (Dataset S1).

Cell density estimates were derived from histological measurements of the number of cells ( n   ) per area unit ( A   ) using the thickness of the sample ( T   ) and the diameter of a single cell ( D   ), based on the formula (12, 13):

[1] $$\rho \;\left[\mathrm{cells}/{\mathrm{cm}}^{3}\right]=\;\frac{n\;[cells]}{A\;[cm^{2}]\;\times \;(T+D)\;[cm]}.$$

Volumetric density was then converted to density per gram of tissue by multiplying by a reference-specific density for tissue of 1.03 g/mL, except for adipose tissue (0.91 g/mL). Differences in tissue-specific density are negligible compared to other uncertainty factors in the analysis.

Relative abundance data, such as flow cytometry results, were incorporated based on cell density estimates or absolute counts of a general cell population. For example, skin macrophage density was estimated based on the fraction they form of nucleated cells in the dermis up to a depth of 300 μm (4, 14).

We constructed a reference for the total mass of different body systems and organs using estimates from ICRP 2002 (11). Furthermore, we categorized the tissues and organs into broad groups based on assumed similarities in immune cell presence. These groups include bone marrow, lymphatic system, blood, barrier epithelial organs [such as the gastrointestinal (GI) tract, skin, lungs, and airways], other epithelial organs, striated muscle, adipose tissue, other connective tissue (including peripheral lymph vessels), the central nervous system, and extracellular fluids and matrix.

For some instances, like the gut and skin, research has indicated the densities in a specific tissue section, such as the epithelium or the dermis. In such cases, we calculated the average density for the entire tissue using approximations of the masses of the various compartments.

**Fig. 1.**
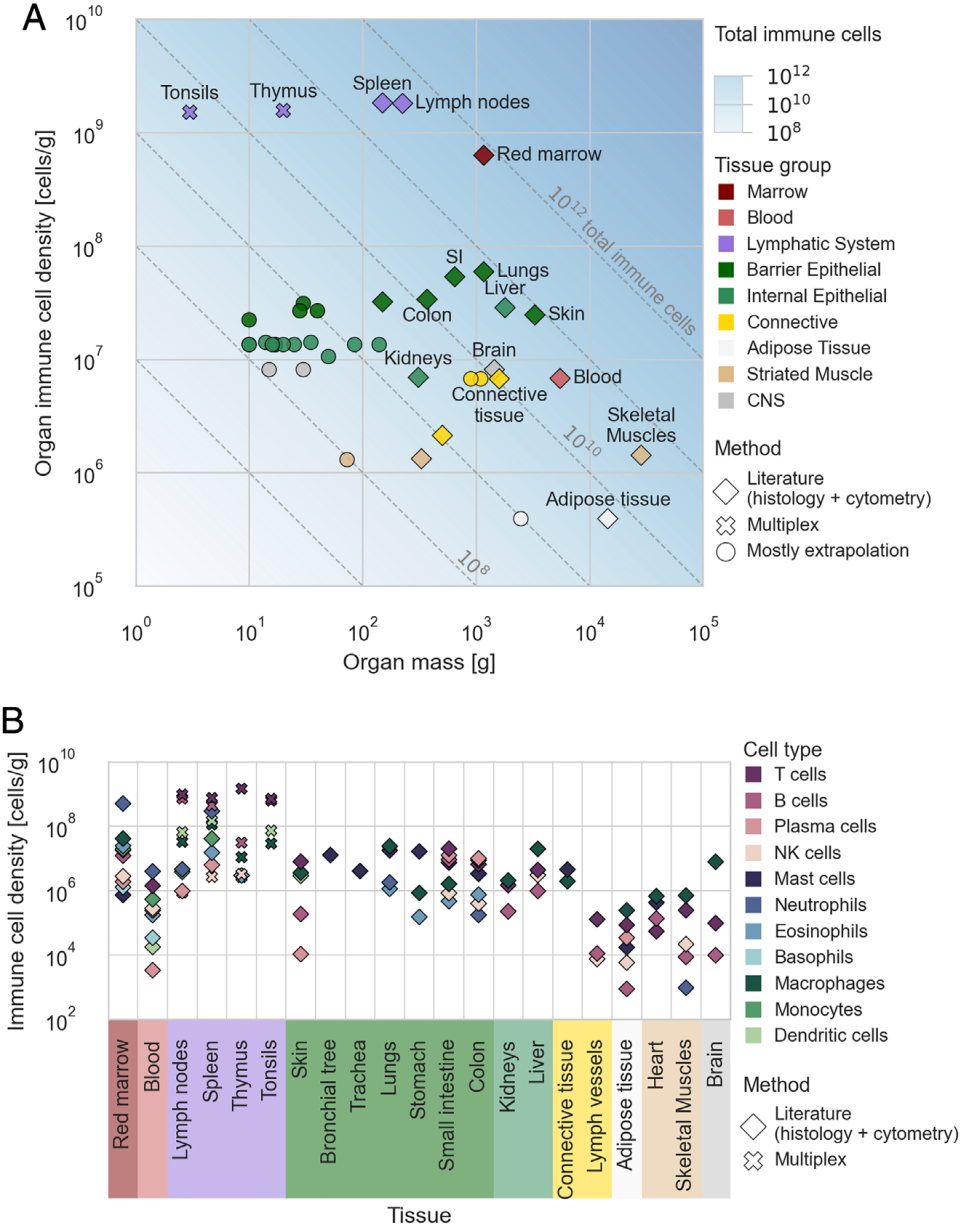
The number of immune cells throughout the body. Cellular densities of immune cell types were estimated based on the literature and multiplexed imaging data. (*A*) The total immune cell density of tissues versus their total mass. Both axes are plotted on a log scale. The diagonals represent the total number of immune cells in the organs. The shape of the markers represents the primary method of the density estimate. Large organs and organs with a high immune cell population are annotated. Tissues without annotation include yellow marrow, cartilage, pancreas, bronchial tree, adrenal, gallbladder, and more (see Dataset S2 for a complete list of the organs and their densities). SI = small intestine. (*B*) Specific immune cell type densities of the various organs and tissues. Only literature and multiplex-based estimates (not extrapolations) are shown. The *y* axis is given in a log scale. The tissues and organs are grouped in both panels by similar structure and function.

When human-derived data were unavailable, cell type–specific tissue density was estimated using two approaches: i) extrapolating from data obtained from other mammals such as mice, rats, and monkeys and ii) calculating the average cellular density of similar organs within the same group (geometric mean). The two extrapolations gave similar values, with differences smaller than a factor of 2 (*SI Appendix*, Fig. S8). We used the geometric mean of the two methods for the following estimates. Fig. 1 shows the densities estimated based on the literature and information about the tissues for which we used extrapolation. The inclusion of extrapolated data had little impact on the estimate of the total population of immune cells, which is evident from Fig. 1*A* (the sum of the extrapolated data is at least two orders of magnitude less than the total number of immune cells obtained using other approaches). While macrophages and lymphocytes are present in most tissues in the body in substantial amounts, rare immune cell types, such as eosinophils and mast cells, are known to only have a significant presence in specific tissues (15, 16). We used the qualitative literature for these tissue-specific immune cells to determine their abundance in the relevant tissues and did not extrapolate the cell density to other tissues. For example, eosinophils reside mainly in the gastrointestinal tract, bone marrow, spleen, lymph nodes, and thymus, with minimal infiltration to other tissues (15). Mast cells can be found in connective tissues and in the lamina propria of barrier epithelial tissues, with a minimal population in the bone marrow (16). Basophils reside in the bone marrow and the blood (16). Hence, the densities of eosinophils, mast cells, and basophils were not extrapolated to additional tissues.

Finally, we combined the immune cell density and mass estimates for each tissue to derive the overall number of cells of each cell type present in each tissue.

#### Multiplexed imaging–based density estimate.

Methods like multiplexed imaging enable the measurement of multiple proteins or targets in situ in a single experiment (17–19). This provides a more detailed and precise understanding of the immune cell population and its distribution in sampled tissues. Through the simultaneous analysis of multiple molecular targets, these techniques offer a snapshot of the diverse immune cell population with high resolution. Therefore, they can be used to determine the density of multiple immune cell types at a high resolution.

Liu et al. (20) provided multiplexed data for secondary lymphoid organs, including the spleen, lymph nodes, thymus, and tonsils. The authors assigned each cell found in the analyzed tissues to a specific cell type based on the 16 markers analyzed by MIBI-TOF. Where applicable, we filtered the data to include only control disease-free lymphoid organs and conducted a quality check on the number of cells in each sample, removing any outlier samples (defined by a z-score over 1.96). Next, we calculated the density of each cell type for each sample using formula [**1**], taking into account the area of the sample (400 × 400 μm^2^) and an effective thickness equivalent to the diameter of a single cell [approximately 7.5 to 17 μm according to specific cell types (16, 21–23); the thickness of the MIBI-TOF beam was assumed to be negligible]. We aggregated the samples by patients and used their geometric mean as the reference density, with uncertainty estimates based on the variation of values within and between patients. The results of our analysis are presented in *SI Appendix*, Fig. S1.

#### Deconvolution based on methylation atlas.

In recent years, researchers developed deconvolution methods for estimating the relative abundance of specific cell types in a sample based on prior knowledge of their genetic or epigenetic signatures. We focused on methylation-based deconvolution data that Loyfer et al. (24) analyzed to validate our immune cell tissue population estimates.

Methylation-based deconvolution can detect different cell types based on their unique methylation patterns and is, therefore, a potentially reliable method for estimating the immune cell population of a tissue. CpG islands, which are typically regulatory regions upstream of many genes, are either methylated or unmethylated. This can induce gene expression variance. By deciphering the methylation patterns of a tissue sample, it is possible to identify cellular subpopulations by their distinct methylation patterns, including immune cells.

In their study, Loyfer et al. examined previously published methylome samples and used an atlas of demethylated cell-type specific signatures to deconvolve their cell-type composition to give the relative frequencies of cell types in different tissues. We obtained the Loyfer et al. dataset of relative frequencies for the derivation of tissue immune cell populations. The dataset contained the proportion of five groups of immune cell types, in various granularity, depending on the methylome signature they obtained. Lymphocytes were partitioned into B cells, T cells, or NK cells. Other immune cell groups were classified as either granulocyte (assumed to correspond to the signatures of neutrophils, eosinophils, mast cells, and basophils) or macrophage + monocyte (denoted by us as nongranulocyte myeloid).

We translated the relative frequency estimate for each cell type into an absolute number using an anchor cell type for which an estimate of its absolute total cell number in this tissue already exists. The absolute number of immune cells ( $N_{\mathrm{im}}$ ) was then estimated using the equation:

[2] $$N_{\mathrm{im}}=\frac{P_{\mathrm{im}}\cdot N_{\mathrm{an}}}{P_{\mathrm{an}}},$$

where the absolute number of anchor cells is given by ( $N_{\mathrm{an}}$ ) and the relative fractions of the immune cell of interest and the anchor cell are given as ( $P_{\mathrm{im}}$ , $P_{\mathrm{an}}$ accordingly).

The anchor values were obtained from Sender and Milo 2021 (4), which contains estimates of the absolute numbers of many different cell types throughout the human body. Tissue samples taken from the same tissue were clustered and averaged to assess the distribution of immune cells throughout different organs. Uncertainty estimates were derived based on the variation between samples via error propagation.

#### Integration of the three methods.

We compared the results obtained by the three methods (literature, multiplexed imaging, and methylome-based deconvolution). Since the estimates derived from literature-based histology were the only ones available for most tissues, they were used as the comparison baseline. The results from the different methods match well wherever the data overlap (*SI Appendix*, Figs. S2–S4). We combined the results from literature-based histology and multiplexed imaging by Inverse-variance weighting in log space and estimated the uncertainties using lognormal parametric estimates. The multiplexed imaging method, which provides high-resolution data, was given greater weight in estimating results for the lymphoid organs. The methylation-based deconvolution results showed relatively high variation and served only as validation for the results derived from the literature.

### Validation by Tabula Sapiens Histological Estimates.

In the context of the Tabula Sapiens project (24), pathologists examined hematoxylin and eosin-stained sections from two donors to assess the proportions of cell types in four compartments: endothelial, epithelial, stromal, and immune. We combined their relative abundance estimates with prior absolute cell number estimates (4) to determine the absolute number of cells present in the immune compartment of the given tissue using Eq. **2**. *SI Appendix*, Fig. S5 summarizes the results from this analysis and compares them to our current estimates of immune cell populations.

### Cellular Mass Estimate.

We obtained estimates for the volume of various cell types by searching through Google Scholar using keywords related to specific immune cell types along with variations of keywords such as “cell,” “volume,” “size,” “diameter,” and “mass.” We also referred to Immunology textbooks (16, 25) for reference estimates on cell sizes. Many estimates were given as ranges for possible cell diameters, so we calculated the cell volume assuming a spherical shape based on the mean diameter. We considered a log-normal distribution for cells with a wide range of diameters and used the geometric mean as the representative diameter. Estimates from blood smears tended to be higher, so we applied a correction factor of 0.7 to account for the potential bias toward overestimating cell sizes. Most estimates were obtained from human samples, but we also collected values from rodents and labeled them accordingly in cases where data were limited.

We aggregated estimates for cell volumes assuming a log-normal distribution, using the geometric mean as the representative volume and the SE in log space as the uncertainty estimate. *SI Appendix*, Fig. S6*A* summarizes the estimates obtained from the literature for different cell types.

Macrophages exhibited a wide range of sizes that appeared to be influenced by the tissue they reside in refs. 16, 21, 22, and 26. We collected estimates from various tissues and categorized them into two groups: tissues with continuous replenishment from monocytes and those without (27). *SI Appendix*, Fig. S6*B* shows the estimates of macrophage sizes by tissue. When possible, we used tissue-specific estimates for macrophage volumes. Otherwise, we used aggregated estimates based on whether monocytes replenish the tissue macrophage population.

We estimated the cell mass by multiplying the volume by a constant specific density of 1.07 g/mL (28). The variation in specific density among immune cells is slight, around 0.02 g/mL (28), and thus negligible compared to the uncertainty in volume estimates.

### Uncertainty Estimates.

We collected or calculated the SE for each value used in our analysis (Dataset S1). The values for cellular density varied significantly, spanning several orders of magnitude for different tissues and cell types. The variation between estimates for a specific tissue and cell type combination could also be considerably high. They could reach an order of magnitude in particular cases due to a combination of differences in measurement methods and biological differences among the tested individuals. In this regard, it is essential to notice that some primary sources had low sample numbers. Due to the large variation between and within tissues, the uncertainty was most accurately represented as a multiplication factor of error (i.e., the uncertainty of a variable with lognormal distribution). To facilitate error propagation, we transformed all values and corresponding errors to be expressed in terms of multiplication error by fitting a lognormal distribution to the normal distribution describing the value and its uncertainty. Thus, we modeled the uncertainty around the value as a random variable x with a lognormal distribution with a shape parameter of ${s=ln(f}_{\mathrm{error}})$ , where $f_{\mathrm{error}}$ is the multiplication error factor. For example, an $f_{\mathrm{error}}=2$ means that there is a 68% probability (one sigma) that the true value is between half and double the given value. By definition, the shape parameter describes the SE of the exponential transformed random variable, defined as $e^{x_{\mu }}$ that is distributed normally.

To propagate the multiplication error of two values with multiplication error, we performed an analytical error propagation using the formula:

[3] $$f_{x\cdot y}=e^{\sqrt{{ln(f_{x})}^{2}+{ln(f_{y})}^{2}}}.$$

This formula is based on the fact that the multiplication of two lognormal variables also follows a lognormal distribution, with a shape parameter equal to the root of the sum of the squares of the original shape factors.

We used bootstrapping to calculate error propagation for the summation of variables with nonlinear uncertainty. Specifically, we drew 1,000 samples from the distribution that described the uncertainties of the values.

Typically, uncertainties associated with values obtained from various sources (e.g., literature studies, methods, etc.) are uncorrelated, making them easy to propagate. However, in some instances, we anticipate that the errors may be correlated, such as when extrapolating density values to multiple tissues. We factored in the potential bias during our calculation, resulting in broader uncertainty for the total values.

## Results

The cellular density data we extracted from the literature (*Materials and Methods*) are presented in Fig. 1*B*. The cellular density is depicted for each cell type and tissue. The tissues are grouped by similar composition and functions. Fig. 1*A* shows the total immune cell density per organ by summing all the cell types belonging to the immune system (*Materials and Methods*). As the *x* axis presents the mass of the organs and tissues, the diagonals represent the total number of immune cells in the tissue.

We observed the highest immune cell densities within the lymphatic system and bone marrow, where the organs consist primarily of immune cells. The immune cell density is similar across all epithelial organs (both barrier and internal) and one order of magnitude lower than that of the bone marrow. The proportion of a specific immune cell type may vary by an order of magnitude across the different epithelial tissues. The densities of macrophage, T, and B cells vary in the range of 10^6^ to 10^7^ cells per gram across these tissues. Plasma cells and eosinophils mainly reside in the gastrointestinal tract, while mast cells reside primarily in connective tissues and epithelial organs’ lamina propria.

Adipose tissue and skeletal muscle tissues comprise about 75% of the body’s cellular mass but hold only 0.2% of the body’s total cell count (3). The total cellular density in these tissues is thus lower than epithelial tissue by several orders of magnitude owing to large cell size. Similarly, the immune cell density in adipose and muscle tissue is up to two orders of magnitudes lower than in epithelial tissue. Nonetheless, the total immune cell population within these tissues is similar to that of epithelial tissues due to their overall high tissue mass.

**Fig. 2.**
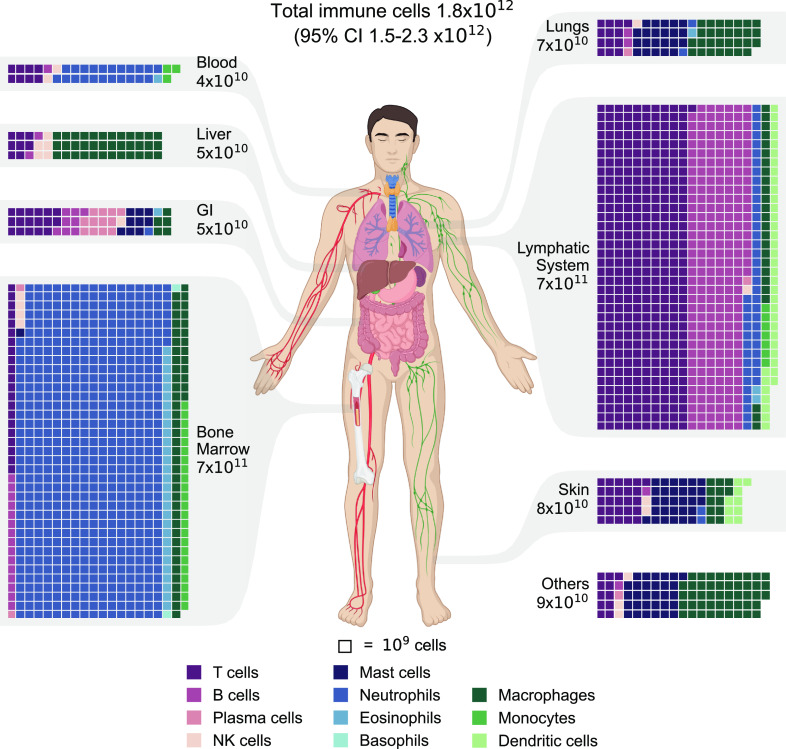
The distribution of immune cells in the human body. Estimates of immune cell populations by cell type and tissue grouped by primary tissues and systems. The tissues are displayed via a chart of the human body. A waffle chart depicts the distribution of immune cells in each tissue, with each square representing a population of 10^9^ cells. To facilitate the presentation, the populations were rounded to multiples of 10^9^. The total population of each tissue is shown with one significant digit. Throughout all the figures, cell types are color-coded for ease of reference. GI = gastrointestinal tract. Other tissues and organs include the brain, heart, adipose tissue, skeletal muscles, kidneys, etc.

We integrated the tissue-specific immune cell densities together with the mass of the organs for the reference human (11) to estimate the distribution of the total number of immune cells across the various tissues in the human body. We compiled the estimates according to the major organs and systems, as presented in Fig. 2. We estimate there to be a total of ≈1.8 × 10^12^ immune cells in the reference person’s body (95% CI 1.5 to 2.3 × 10^12^). Most immune cells reside in the bone marrow and the lymphatic system, comprising 40% and 39% of all cells, respectively. Skin, lungs, and the gastrointestinal tract each comprise 3 to 4% of all immune cells in the human body, while only ≈2% are found in the blood. Even though the blood contains ≈90% of the cells in the body (3, 4), only 0.1% are white blood cells, while the rest are red blood cells and platelets.

The results in Fig. 2 demonstrate a diverse composition of immune cells across different tissues. The bone marrow contains ≈7.4 × 10^11^ cells (95% CI 6–9 × 10^11^), with a dominant presence of neutrophils, accounting for approximately 80% of the population. Similarly, the lymphatic system harbors ≈7.2 × 10^11^ immune cells (95% CI 5–10 × 10^11^), with lymphocytes being the prevailing cell type, constituting nearly 85% of the total. The digestive system also exhibits a relatively high lymphocyte population, with lymphocytes comprising approximately 70% of the GI tract’s 5 × 10^10^ immune cells (95% CI 3–9 × 10^10^). Additionally, mast cells contribute significantly to the immune cell population of the digestive system, comprising about a quarter of it. Mast cells also play a substantial role in the immune cell populations of the lungs and skin, accounting for roughly 30% of the total immune cells in each (7 × 10^10^ for the lungs and 8 × 10^10^ for the skin). Macrophages represent a minor fraction of immune cells in tissues such as the bone marrow, lymphatic system, and gastrointestinal tract. However, in the liver, they contribute approximately 70% of the immune cell population (5 × 10^10^, 95% CI 4–7 × 10^10^), and in the lungs, they account for around 40% of the total immune cell population.

**Fig. 3.**
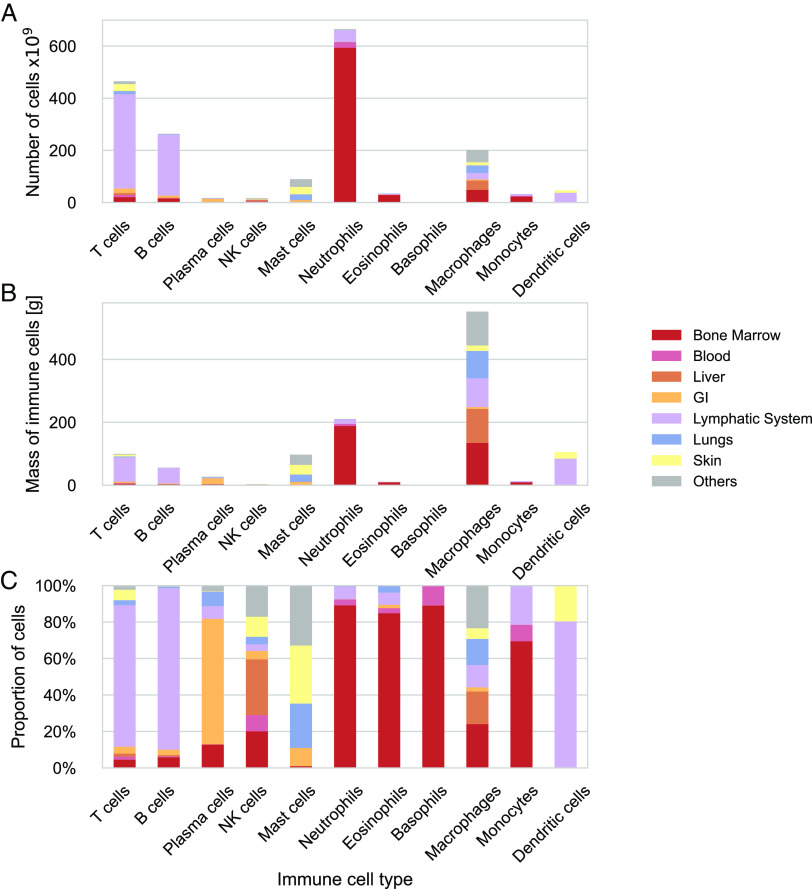
Cell type–specific tissue distribution of immune cells in the human body. Estimates of immune cell populations by cell type and tissue grouped by primary tissues and systems. For each cell type, the distribution across the systems is depicted in the absolute number of cells (*A*), absolute mass of cells (*B*), and relative number of cells (*C*).

Fig. 3 displays a complementary picture from the perspective of the overall distribution of immune cell types in the body as a whole. It shows the cell-type-specific distribution across the tissues. T cells, B cells, and dendritic cells are primarily located in the lymphatic system, while around 70% of the plasma cells are in the gastrointestinal tract. Neutrophils, eosinophils, monocytes, and basophils are mainly found in the bone marrow, with less than 10% in the blood. In contrast, mast cells, NK cells, and macrophages are tissue-resident cells without a dominant distribution in any system.

To validate the estimated numbers of immune cells in various tissues, we used data from a deconvolution approach based on a methylation atlas (29). Using the cell type proportions within tissues estimated by Loyfer et al., we calculated the total number of specific immune cells in these tissues (see the *Materials and Methods* section). Our results show a relatively close correspondence between the estimates generated by the literature-based method and those derived from the methylome-based data (*SI Appendix*, Figs. S3 and S4). Given the considerable uncertainties of the estimates, there is agreement in most cases. Notably, a better agreement is observed for lymphocyte estimates, while the methylome-based method tends to underestimate the granulocyte population.

**Fig. 4.**
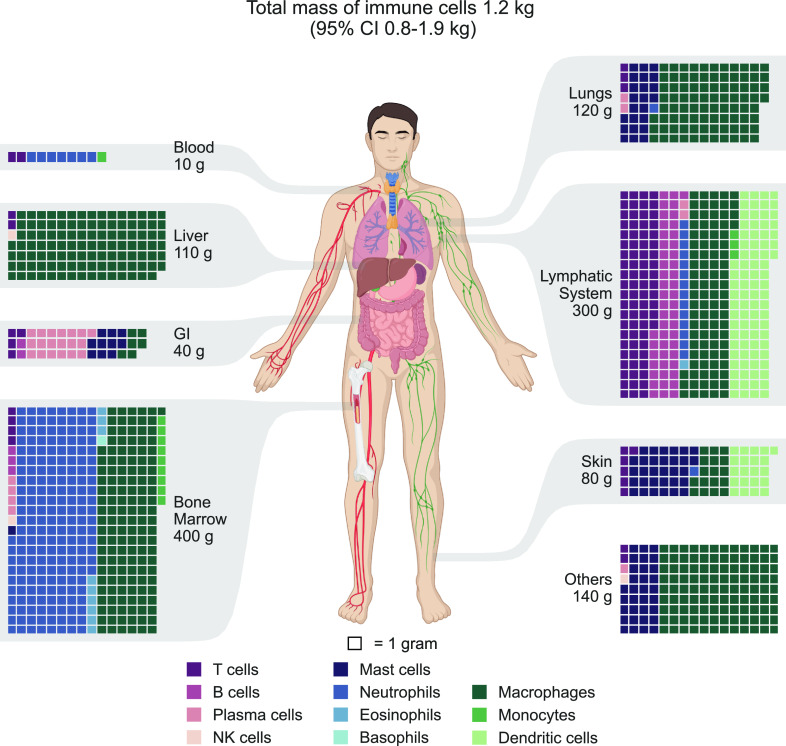
The distribution of immune cell mass in the human body. Estimates of immune cell mass were integrated based on the cellular mass and population. The estimates were grouped by cell type by primary tissues and systems. The tissues are displayed via a chart of the human body, while a waffle chart is used to depict the distribution of immune cell mass in each tissue, with each square representing 1 g of immune cells. To facilitate the presentation, the populations were rounded to multiples of 1 g. The total immune cell mass of each tissue is shown with one significant digit. Throughout all the figures, cell types are color-coded for ease of reference. GI = gastrointestinal tract. Other tissues and organs include the brain, heart, adipose tissue, skeletal muscles, kidneys, etc.

Data on the mean mass of specific immune cell types are limited. To obtain a representative cell mass for each cell type, we collected data on the sizes and volumes of immune cells from the literature and integrated them, as presented in *SI Appendix*, Fig. S6. By combining the estimates for cellular distribution and mass, we obtained an estimate for the distribution of immune cells by mass, as shown in Fig. 4. Our estimates suggest that there are a total of 1.2 kg of immune cells in the body (95% CI 0.8–1.9 kg). As with the distribution by numbers, the bone marrow and lymphatic system contain most of the cellular mass, accounting for 30% and 27% of total mass, respectively. The lungs and liver each account for approximately 10% of the immune cell mass, while only about 1% is present in the blood. The gastrointestinal tract, the skin, and other tissues contain the remaining 23% of the total immune cell mass.

Table 1 summarizes each cell type’s contribution to the total number of immune cells in terms of numbers and mass. Lymphocytes comprise 40% of the immune cells in the body, with approximately 7.6 × 10^11^ cells (95% CI 5–12 × 10^11^). T cells account for 60% of lymphocytes, while B cells account for about a third. Granulocytes constitute 43% of the immune cells, with 8 × 10^11^ cells (95% CI 7–10 × 10^11^). Neutrophils comprise over 80% of these cells, most of which reside in the bone marrow. The myeloid cell types account for the remaining 15%, most of them being macrophages distributed throughout the various tissues.

**Table 1.** The total population of immune cell types in the body

Cell type	Total number	95% CI	Total mass [g]	(95% CI)
T cells	5 × 10^11^	3–7 × 10^11^	100	60–150
B cells	3 × 10^11^	2–4 × 10^11^	60	40–90
Plasma cells	2 × 10^10^	0.5–6 × 10^10^	30	4–170
NK cells	2 × 10^10^	0.9–4 × 10^10^	4	2–8
Mast cells	9 × 10^10^	6–14 × 10^10^	100	40–200
Neutrophils	7 × 10^11^	5–8 × 10^11^	200	160–300
Eosinophils	4 × 10^10^	2–8 × 10^10^	10	5–20
Basophils	2 × 10^9^	0.4–7 × 10^9^	0.7	0.2–3
Macrophages	2 × 10^11^	1.3–3 × 10^11^	600	300–1,400
Monocytes	3 × 10^10^	2–4 × 10^10^	13	9–20
Dendritic cells	5 × 10^10^	3–8 × 10^10^	100	40–300
Total	1.8 × 10^12^	1.5–2.3 × 10^12^	1,200	800–1,900

The contribution of the various immune cell types is summarized in terms of numbers and total mass. Cell types are arranged according to their respective groups (analogous to the layout in all figures). Estimates are rounded to the first significant digit. The 95% CI is provided for each estimate.

**Fig. 5.**
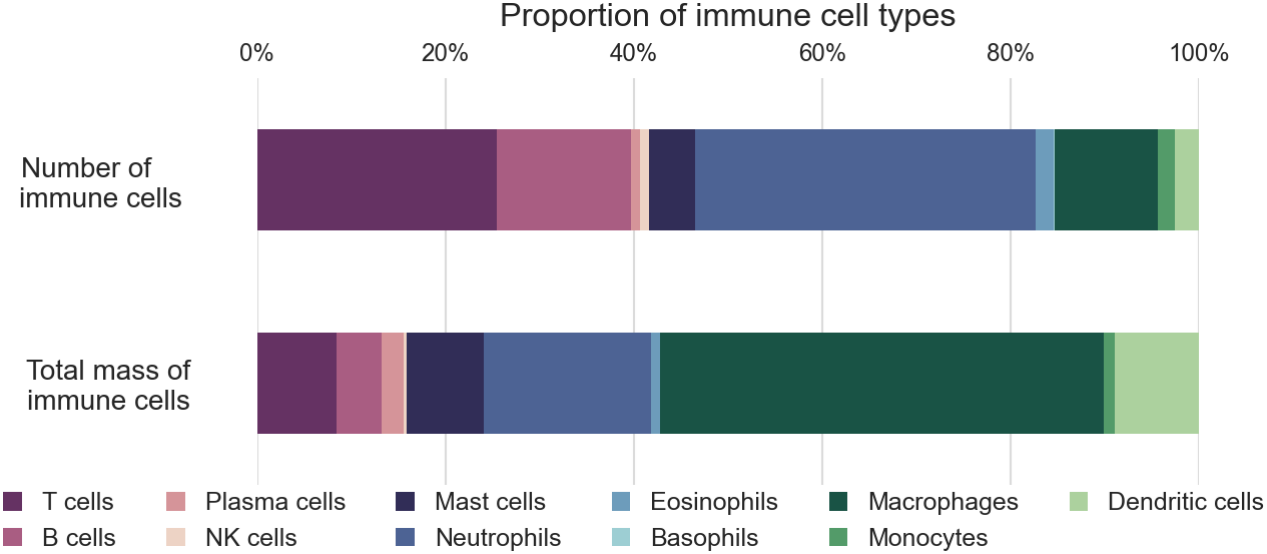
The proportion of immune cell types in the body by number and mass.

The distribution of immune cell types by mass differs significantly from their distribution by number, mainly due to the considerable variation in cell sizes (Fig. 5). While most immune cells are relatively small and weigh a few hundred picograms, macrophages are significantly larger, with a weight of a few nanograms. Consequently, lymphocytes represent only about 15% of the immune cell mass, equivalent to less than 200 grams (95% CI 100–300 g). On the other hand, Macrophages weighing 600 grams (95% CI 300–1,400 grams) and dendritic cells weighing 100 grams (95% CI 40–300 g) account for 49% and 9% of the total immune cell mass, respectively, mainly due to their larger cell size. Granulocytes comprise around one-quarter of the immune cell mass, weighing approximately 320 grams (95% CI 240–420 grams), with two-thirds originating from neutrophils located primarily in the bone marrow.

Thus far, we have used the standard reference human (male) weighing 73 kg (11). Using the tissue-specific density data, which is independent of sex and age, we derive the distribution of immune cells in a reference female weighing 60 kg and a reference child aged 10 years weighing 32 kg by considering their organ reference masses (11). We estimate that there are 1.5 × 10^12^ immune cells in the reference female, with a mass of about 1 kg. The reference child harbors 1 × 10^12^ immune cells weighing about 600 g. The distribution by cell type and tissue is very similar to those of the reference male, as the organ reference mass of many of the tissues scales linearly with the mass of the individual across the different sexes and ages. As the reference mass of the lymph nodes is not given for children or adult females, we interpolated it linearly from that of the reference adult male.

## Discussion

Here, we have estimated the total number, mass, and distribution of immune cells in the human body. These estimates can serve as a base for answering some basic quantitative questions regarding the immune system that have so far been left unanswered. For example, which is the largest immunological organ in the human body? The gastrointestinal tract is commonly stated to comprise the majority of the immune cells (5) or at least of the lymphocytes (30, 31). Our analysis suggests that the most significant immunological organs are the bone marrow, lymph nodes, and spleen. The gastrointestinal tract contains about 3% of the cells of the immune system and around 5% of the lymphocytes; thus, most of the lymphocytes do not reside in the gut, as Ganusov and De Boer concluded (7). However, as shown by ref. 10, the gut is home to ≈70% of the plasma antibody-producing cells in the body, and thus, it is the largest compartment of the body concerning the humoral system (Fig. 3). A similar situation exists for NK cells, with a dominant liver population (≈30% of the NK cells).

Prior computational studies either had a specific focus on certain tissues or immune cell types (1) or examined immune cells within a broader analysis, but some details were lacking (2–4). When comparing the current findings to previous research, it is evident that Bianconi et al. (2) overlooked a significant portion of immune cells by only examining lymphocytes in the blood and not distinguishing between cells in the bone marrow. Sender et al. (3, 4) provided an estimate for the total number of immune cells similar to that of the current analysis. The current distribution is more variable, with a higher proportion of myeloid cells resulting in a greater overall mass. Trepel’s (1) research centered on lymphocytes and their distribution throughout the human body, relying on data from rodents and extrapolating to humans. Trepel estimated that there are almost 5 × 10^11^ cells, with two-thirds in the lymphatic system, 10% in the bone marrow, and 2% in the blood. Our current analysis showed that human cell densities are comparable to rodents in most tissues. Our overall estimate of 7.6 × 10^11^ lymphocytes (95% CI 4−12 × 10^11^) is similar to Trepel’s extrapolation but with a larger population of lymphocytes in the lymphatic system, as multiplex data revealed a higher density than rodent data. In their study, Storek et al. (32) utilized stem cell allograft infusion to quantify the circulating lymphocytes via cellular kinetics in patients with hematologic malignancies, which resulted in an estimate of approximately 10^12^ cells. Given the uncertainty of the method and their focus solely on patients with hematologic malignancies, this corresponds with our findings.

The distribution of immune cells in terms of numbers and mass presents an intriguing juxtaposition. Around 75% of immune cells are lymphocytes and neutrophils, which are among the smallest cells in the body, weighing only a few hundred picograms. Thus, their cellular mass only accounts for about 30% of the total immune cell mass. On the other hand, macrophages, dendritic cells, and mast cells, which are 3 to 10 times larger, make up less than 20% of immune cells but contribute to over 60% of the immune cell mass.

Analysis by mass also highlights the liver as a unique internal organ in terms of the immune system. Our estimate indicates that ≈6% of the liver’s 1.8 kg mass (for the reference human) is occupied by immune system cells. This aligns with the notion that beyond its metabolic and detoxification functions, the liver serves as a front-line immune barrier, countering constant exposure to foreign antigens, primarily originating from the gut (33).

We sought an independent methodology for quantifying immune cell densities or total counts to validate our estimates. We found three data sources for such validation, each with limited scope. Multiplexed imaging data for lymphoid organs served as the primary validation for the density estimates in these tissues (*SI Appendix*, Fig. S2). Human-based data on cell densities in these tissues were notably limited. Some of the estimates were derived from animal data, introducing substantial uncertainties when extrapolating these findings to humans. We combined the literature and multiplexed imaging data to derive an integrated estimation of the density and its uncertainty. Another source for validation was methylome deconvolution data. Accounting for the uncertainties of the deconvolution method and our literature-based estimate, there was a general agreement between both approaches (*SI Appendix*, Figs. S3 and S4). Notably, we observed a stronger concordance for lymphocyte estimates, while the methylome-based method underestimates the granulocyte population. This could be due to the propensity of dissociation methods to eliminate a substantial portion of neutrophils. In a few instances, there seems to be a bias in the deconvolution-based results. For example, in the pancreatic immune cells, the deconvolution-based results are an order of magnitude higher than the literature-based results. This might be due to the precision of the deconvolution analysis for the small fraction of beta cells used as anchors or due to the estimate of the total number of beta cells used as anchors. An alternative explanation is that the literature-based results are underestimated because they are based on an extrapolation from similar tissues. The last method for validation was based on an independent histological analysis of the fraction of immune cells by the Tabula Sapiens consortium (*Materials and Methods*). These data were available only from two patients around age 60 without separation into individual cell types. Nonetheless, comparison with the current estimates shows a general agreement (*SI Appendix*, Fig. S5), with less than an order of magnitude difference for each tissue, usually favoring the Tabula Sapiens estimate.

The distribution of immune cells in a reference adult female and child was estimated using tissue-specific densities, assuming independence of sex and age due to insufficient data for differentiation between populations. Thus, our estimate for the effect of young age and sex on total immune cell numbers and mass is mainly proportional, driven by the mass differences between the populations. Minor nonlinearities in the masses of reference organs account for slight differences in relative distribution (*SI Appendix*, Fig. S7*B*).

Previous studies investigating the impact of sex on the immune system have identified it as a primary factor influencing overall blood immune cell composition (34, 35). The literature generally agrees that females exhibit more robust immune responses than males, partially attributed to the effects of sex hormones like estrogen and prolactin on B and T lymphocyte maturation and increased B cell antibody production (36). However, more quantitative data are needed to determine differences in immune cell type distribution throughout the body’s tissues. Closing this data gap will facilitate a more nuanced sex-specific estimate.

Turning to the effects of age, we can first look at the populations of immune cells in the bone marrow and lymphatic system, as our results indicate they comprise nearly 80% of the immune cells. While all bone marrow is active after birth, the active fraction declines during childhood. Despite substantial changes in the distribution of active marrow during maturation, the percentage of total body mass represented by active marrow remains relatively stable over several decades (11). The cellular distribution within the bone marrow undergoes distinct changes during the initial year of life and from that point and onward into adulthood stays relatively constant (16). Aging leads to a decline of up to 40% in bone marrow cell numbers and composition (37–41), potentially causing a nearly 20% decrease in the total immune cell population and shifting its distribution.

The immune population of the lymphatic system predominantly resides in the spleen and lymph nodes, with a smaller fraction in the thymus. The spleen mass scales proportionally with body mass in children and adults (11). However, significant variation exists among different adult populations in various countries. Unfortunately, data regarding changes in total lymph node mass are lacking. The thymus grows until puberty and then undergoes partial involution (42), resulting in age-related decrease in weight (11), as well as in lymphocyte density (43, 44). Given the thymus’s relatively small size (less than 10% of adult lymphatic systems), its effect on the overall lymphocyte population during aging is minor.

Aging also affects the immune cell population in other systems. Animal studies demonstrate an age-dependent increase in the macrophage population in the liver and adipose tissues by several fold (45, 46), a similar increase observed in human adipose tissue (47). Age-related trends for lymphocytes, primarily observed in the blood, include a decrease in naive cells and an increase in terminally differentiated cells, contributing to reduced vaccine efficiency (48). However, the overall number of circulating lymphocytes changes minimally (49). A recent review (50) summarizes data from mice and humans single-cell studies, revealing shared trends: with age, most circulating immune cell populations decline, except for monocytes that increase in number. These general trends align with a longitudinal study of immune cell composition (51), demonstrating specific age-related composition changes as a meaningful metric of immune age, predicting all-cause mortality.

In conclusion, age-related changes lead to a substantial decrease in bone marrow immune cells and potentially in other tissues, as observed in the blood. Similarly, it causes an increase in myeloid cells in other tissues, such as adipose tissue and liver. However, further characterization is necessary to quantify the effect of aging on immune cell distribution precisely.

Looking through a broader lens on the variation of immune system composition within healthy individuals, we turn to recent studies that mainly focus on the blood due to its accessibility for quantitative measurements. These investigations utilize techniques such as genome-wide association or twin studies to assess the influence of genetic and nongenetic factors on immune cell and immune-related protein composition. While there is a general agreement on considerable interindividual variation, its source remains inconclusive. Some studies emphasize genetic factors contributing to the variation (52, 53), while others highlight sex and nonheritable influences, such as age, smoking, and exposure to pathogens like Cytomegalovirus (CMV) (34, 35). Given that the blood immune cells constitute a small fraction of all immune cells, further investigation is warranted to explore the impact of these factors on the immune cell distribution throughout the body.

Given that pathogen exposure is recognized as a significant environmental factor influencing the diversity and ubiquity of immune cell populations (52), it is worth exploring its potential quantitative impact. A recent study by Wijeyesinghe et al. (54) investigated this by comparing laboratory mice cohoused with pet shop mice to mice raised under standard conditions. Employing quantitative immunofluorescent microscopy across various tissues, they observed an increase in the frequency of immune cells, contributing to the overall number of cells in the organs. Notably, immune cell populations in tissues such as the liver, intestine, and kidneys exhibited a substantial increase of 25 to 100%. While it is plausible to assume a similar effect in humans, the overall impact on the total immune cell population would likely be limited due to the relatively minor contribution of these tissues to the total immune cell pool.

More generally, changes in the lymphocyte population can occur during an immune response to infection, cancer, or other conditions. Lymphadenopathy, an increase in the size or consistency of lymph nodes (55), can be caused by various factors such as bacterial or viral infections or cancer. The diameter of healthy lymph nodes is typically up to 1 cm. Infections typically cause moderate and localized volume increases. In most cases, nodes that grow beyond a size of 1.5 × 1.5 cm^2^ are malignant (56, 57). Since lymphocytes have the highest cellular density in lymph nodes, we can use this rule of thumb to estimate the increase in lymphocyte numbers during lymphadenopathy. Assuming a normal spherical lymph node with a diameter of 0.5 cm, we arrive at an estimated reference size of around 0.5 cm^3^, which matches previous estimates (58, 59). If we assume that during lymphadenopathy, the diameter of a lymph node increases by a factor of 2 to 2.5, the volume increases by a factor of 8 to 15. However, this enlargement is limited to only a tiny fraction of the numerous lymph nodes in the body. Assuming the effect is localized to up to 10 lymph nodes, which represent approximately 2% of the total lymph nodes in the body, we can expect an overall increase of 20% at most in the total lymph node lymphocytes or 10% in the total lymphocytes. Splenomegaly, an enlargement of the spleen, which can be caused, for instance, by infectious mononucleosis, can increase the volume of the spleen twofold (60), increasing the size of the lymphatic system by over 30%. Therefore, this alteration could increase the total number of immune cells by 10% and the weight of the immune system by 5 to 10%.

In obesity, the weight of the adipose tissue can easily triple. For example, a man with the same height as the reference human weighing 50 kg more will have a body mass index (BMI) of 40 kg/m^2^. A large part of this increase is attributed to the adipose tissue. Studies in mice and humans indicate that the density of adipose tissue macrophages increases linearly in obesity (61). Thus there is an increase of an order of magnitude in the total number of macrophages in adipose tissue. Obesity is a multiorgan disease, and studying the effects of obesity on the size and composition of the immune system requires the consideration of subcutaneous and visceral adipose tissues, macrophage infiltration into skeletal muscle, changes in the composition of the bone marrow (62), and other factors.

The immune system is dynamic and built to respond to changes in conditions, such as the appearance of pathogens. It is hard to quantitatively estimate the effects of health conditions on the number of immune cells and their distribution. During an immune response to a pathogen, neutrophils and monocytes migrate to the location of the infection and interact with the resident macrophages. The distribution of neutrophils is temporarily altered due to the migration of the marginal pools existing in the spleen and bone marrow. This is further altered by an increased production rate of neutrophils in the bone marrow and a prolonged lifespan induced by the immune response (63, 64).

Our analysis comprises numerous components integrated from various sources and methods, each with limitations. We aim to account for our analysis’s different sources of uncertainty rigorously. To achieve this, we have carefully assessed the errors at each stage and propagated them while accounting for potential biases, as described in the *Materials and Methods* section. The two primary sources of uncertainty in our analysis are the number of lymphocytes and the cellular mass of macrophages. The estimate of the lymphocyte densities in the lymphatic system, derived from a combination of multiplex data and histology, dramatically affects the number of lymphocytes. However, the overall estimate has considerable uncertainty due to the scarcity of literature data, particularly from human patients, and the small number of patients in the multiplex data. Furthermore, multiplexed imaging was performed on “control” tissues that were disease-free but might have been obtained from unhealthy patients (often postmortem), which could bias the results. The mass of a single macrophage varies by nearly an order of magnitude across different tissues. Most of the data we obtained on macrophage sizes consist of textbook diameter estimates. As a result, the distribution of macrophage sizes is poorly understood, leading to a large degree of uncertainty in the total mass estimate.

Our analysis operates under the implicit assumption that the uncertainty concerning the mass of organs is insignificant. Therefore, we utilize the values provided for the reference population (11). The most significant impact of this assumption is on the bone marrow and lymphatic system, which are responsible for the majority of immune cells, both in quantity and mass. The ICRP report has exhaustively reviewed the literature regarding the mass of active bone marrow and spleen, so we anticipate they will not introduce significant uncertainty to our calculations. However, our analysis reference mass for lymph nodes is restricted to a prior report (59) and a modeling study (58). Thus, the involved uncertainty could affect our overall lymphocyte estimate by a few tens of percent.

In addition to the sources of uncertainty previously discussed, this analysis is constrained by the limited availability of data and resolution concerning the cellular makeup of many tissues. We address this in a limited manner by utilizing data from high-throughput techniques such as computational deconvolution and multiplex analysis to create detailed snapshots of specific tissues. We are optimistic that such methods have tremendous potential for shedding light on the cellular composition of the human body in similar analyses. Future investigations could employ these and other advanced techniques within a framework to more accurately characterize the cellular composition and its variability across different populations. Including precise, automated size measurements would also contribute to more accurate estimates of cellular mass.

Characterizing the distribution of immune cells in various tissues reveals common trends in tissue organization as well as tissue-specific differences. Thus, our findings highlight the complex nature of the immune system and could lead to the identification of principles that govern its organization.

## Supplementary Material

Appendix 01 (PDF)

Dataset S01 (XLSX)

Dataset S02 (XLSX)

## Data Availability

Liu et al. (20) multiplexed dataset is available on Zenodo: https://doi.org/10.5281/zenodo.5945388 (65). All code is available in Jupyter notebooks at https://gitlab.com/milo-lab-public/distribution-of-immune-cells (66). All other data are included in the manuscript and/or supporting information.
