# Supplementary material for: The total mass, number, and distribution of immune cells in the human body
Source: Proc Natl Acad Sci U S A. 2023 Oct 23;120(44):e2308511120. doi: 10.1073/pnas.2308511120 (PMC10623016; doi:10.1073/pnas.2308511120)
Supplement: Supplementary file 1 — Appendix 01 (PDF) [file pnas.2308511120.sapp.pdf]

PNAS

Supporting Information for

The total mass, number, and distribution of immune cells in the human body

Ron Sender[1], Yarden Weiss[2], Yoav Navon[1], Idan Milo[3], Nofar Azulay[3], Leeat Keren[3], Shai Fuchs[4], Danny Ben-Zvi[5], Elad Noor[1], Ron Milo[1]*

[1] Department of Plant and Environmental Sciences, Weizmann Institute of Science, Rehovot 76100, Israel.
[2] Department of Molecular Genetics, Weizmann Institute of Science, Rehovot 76100, Israel.
[3] Department of Molecular Cell Biology, Weizmann Institute of Science, Rehovot 76100, Israel.
[4] Pediatric Endocrine and Diabetes Unit, Edmond and Lily Safra Children's Hospital, Sheba Medical Center, Ramat Gan 52621, Israel.
[5] Department of Developmental Biology and Cancer Research, Institute for Medical Research Israel-Canada, The Hebrew University-Hadassah Medical School, Jerusalem 91120, Israel
* Corresponding author: ron.milo@weizmann.ac.il

This PDF file includes:

Figures S1 to S8

Other supporting materials for this manuscript include the following:

Datasets S1 to S2

Supplemental figures

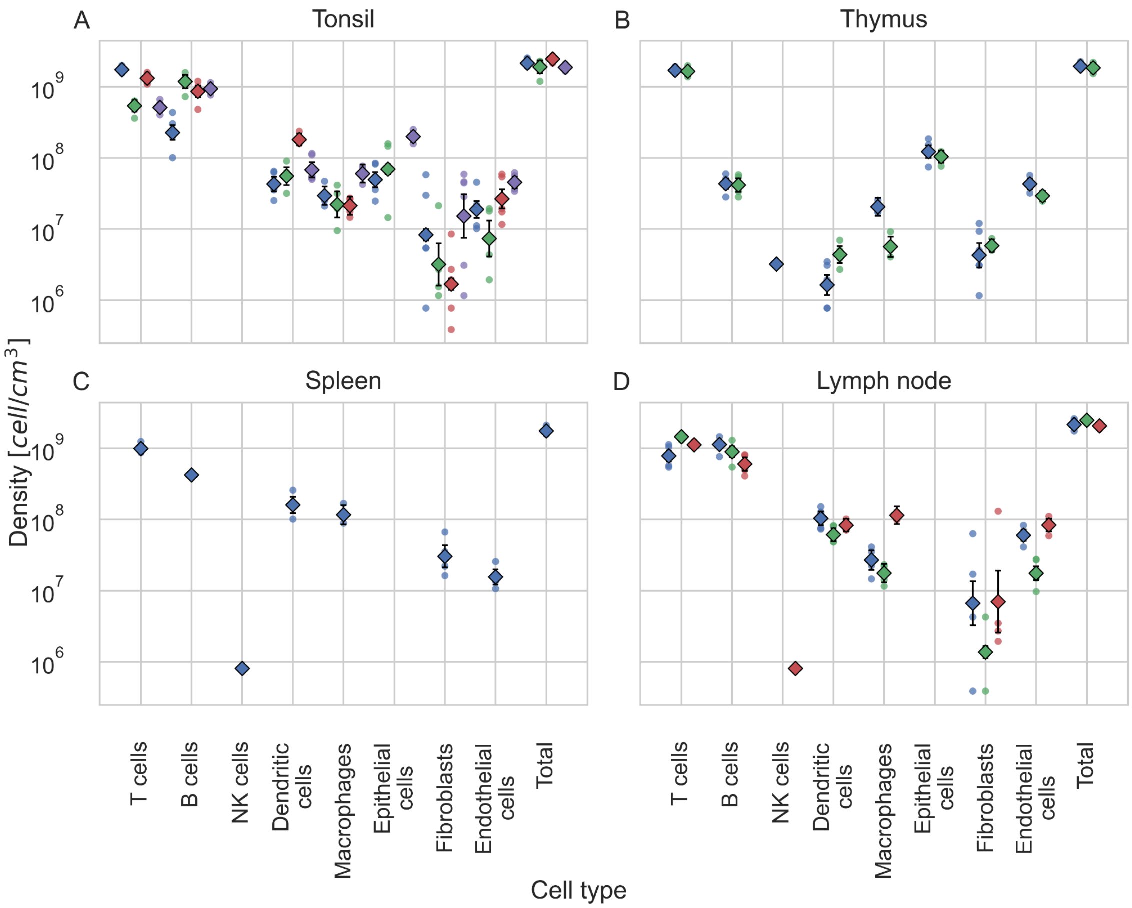

Fig. S1: Multiplex-based estimates for the cellular densities of immune cells in the lymphoid organs. *Estimates for the densities of the various cell types were derived from the data of Liu et al. 2022 (20) (see Methods). Each panel presents a specific lymph organ. The Y-axes are log-transformed. The markers are color-coded according to the patient from which the sample was taken. Each dot represents a specific sample from that patient. The diamond markers depict the geometric means with uncertainty, including the measurement error. Error bars represent 1 standard error of the mean, assuming log-normal uncertainty.*

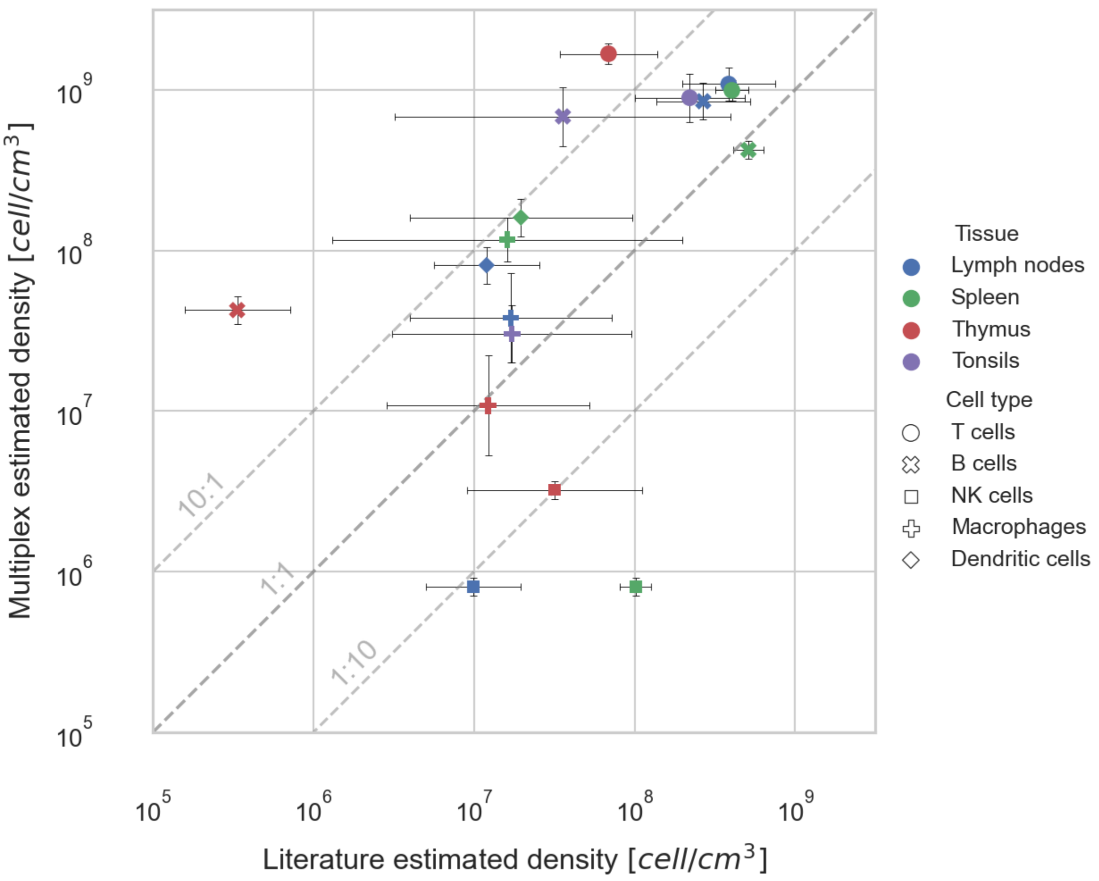

Fig. S2: Comparison of literature-based and multiplex-based estimates for the total number of immune cells in the organs of the lymphatic system. *Literature estimates are based on the integration of flow cytometry and histological measurements. In lymph nodes, there are no direct measurements of densities from humans. Thus, estimates from non-human animals served as a basis for comparison. Multiplex-based estimates were derived from the data of Liu et al. 2022 (20)* (see Methods). Both axes are log transforms. Error bars represent 1 standard error of the mean, assuming log-normal uncertainty. The diagonal lines represent constant ratios between the estimates. Most values are within an order of magnitude difference.

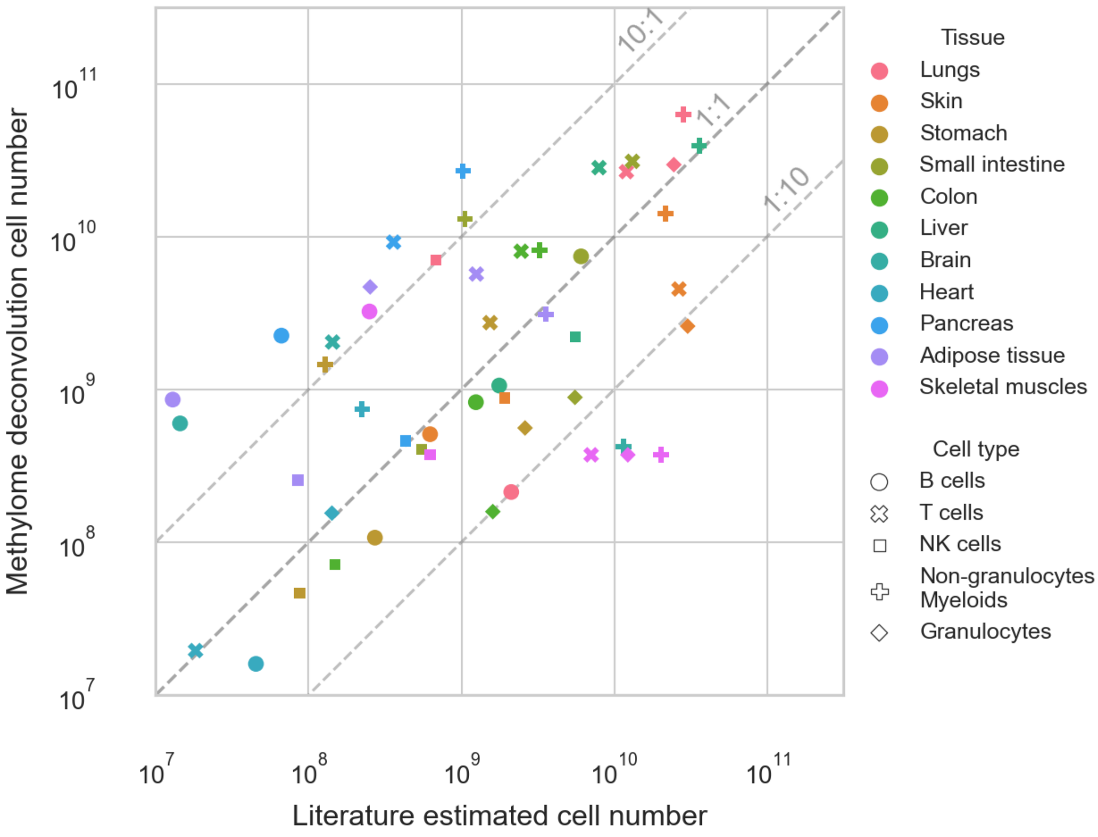

Fig. S3: Comparison of literature-based and methylome deconvolution-based estimates for the total number of immune cells in various tissues. *Literature estimates are based on the integration of flow cytometry and histological measurements. Methylome deconvolution-based estimates were derived from the data of Loyfer et al. 2023 (29)* (see Methods). Cell types (granulocytes, non-granulocytes myeloids) were aggregated to match the methylome-based data. Both axes are log transforms. The diagonal lines represent constant ratios between the estimates. Most values are within an order of magnitude difference.

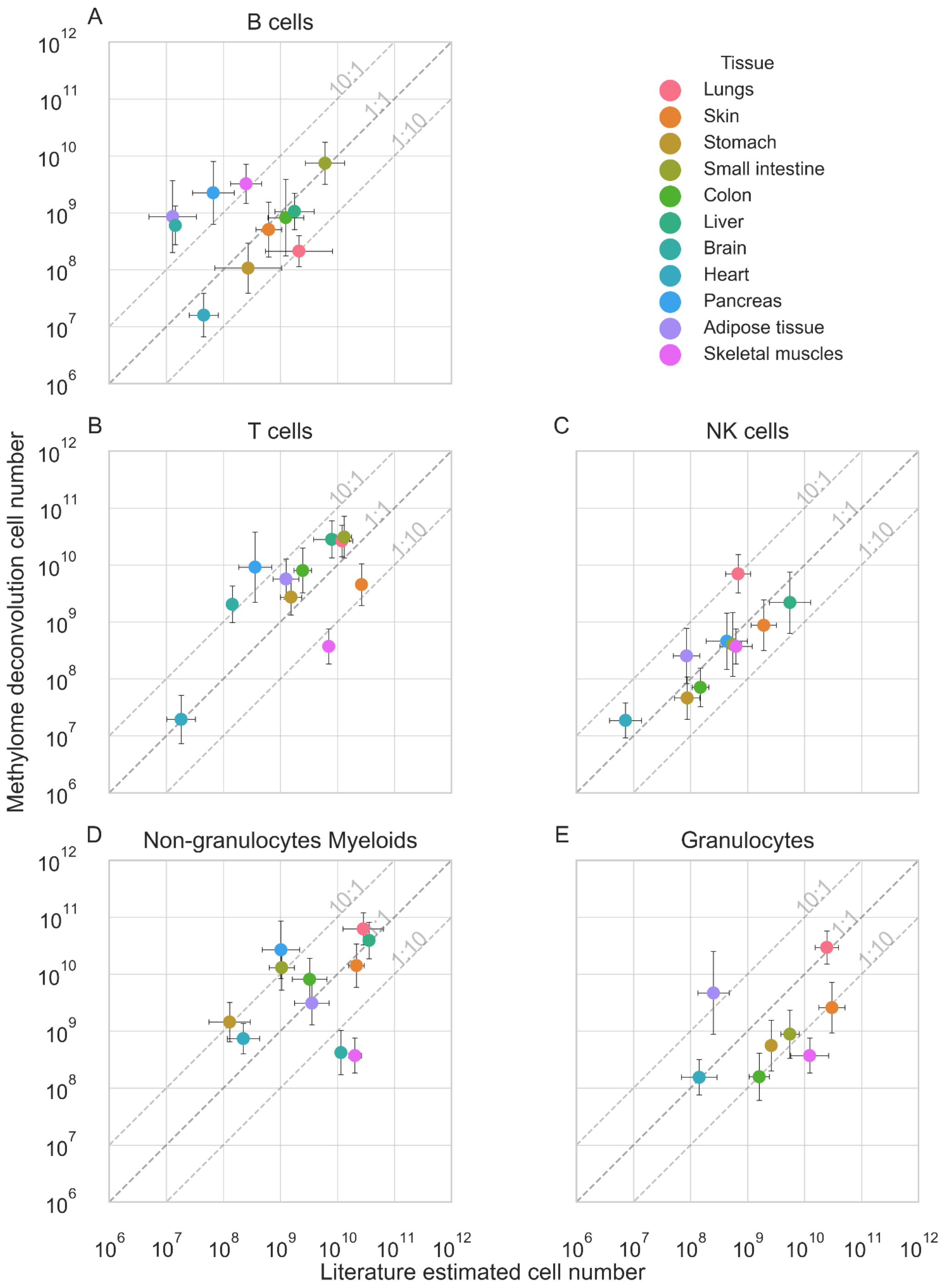

Fig. S4: Comparison of literature-based and methylome deconvolution-based estimates for the total number of specific immune cell types. *Literature estimates are based on the integration of Flow Cytometry and histological measurements. Methylome deconvolution-based estimates were derived from the data of Loyfer et al. 2023 (29)* (see Methods). Cell types (granulocytes, non-granulocytes myeloid) were aggregated to match the methylome-based data. Each panel (A-E) represents a

comparison of a specific cell type. Both axes are log transforms. Error bars represent one standard error of the mean, assuming log-normal uncertainty. The diagonal lines represent constant ratios between the estimates. Most values are within an order of magnitude difference.

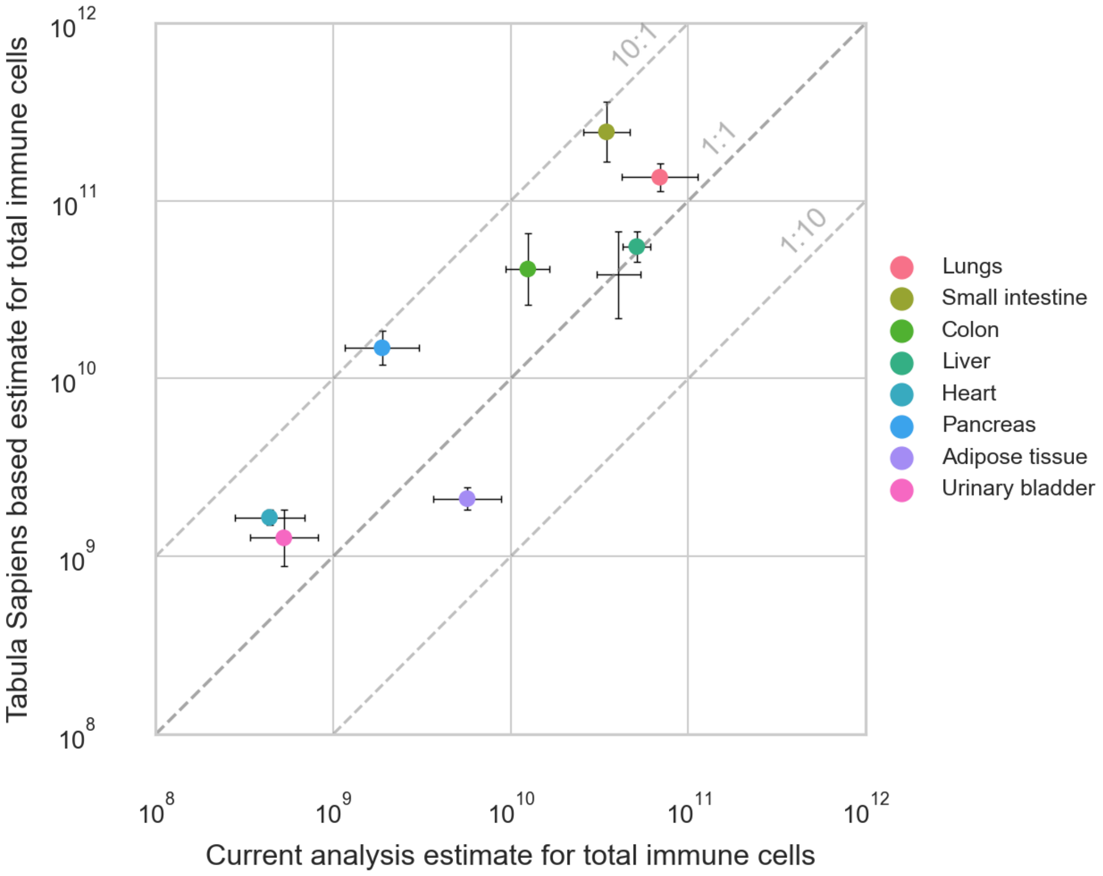

Fig. S5: Comparison of literature-based and Tabula Sapiens histology-based estimates for the total number of immune cell types in different tissues. *Literature estimates are based on the integration of flow cytometry and histological measurements. Tabula Sapiens histology-based estimates were derived from histology data obtained from two donors by the Tabula Sapiens consortium (24)* (see Methods). Error bars represent 1 standard error of the mean, assuming log-normal uncertainty. The diagonal lines represent constant ratios between the estimates. All values are within one order of magnitude difference.

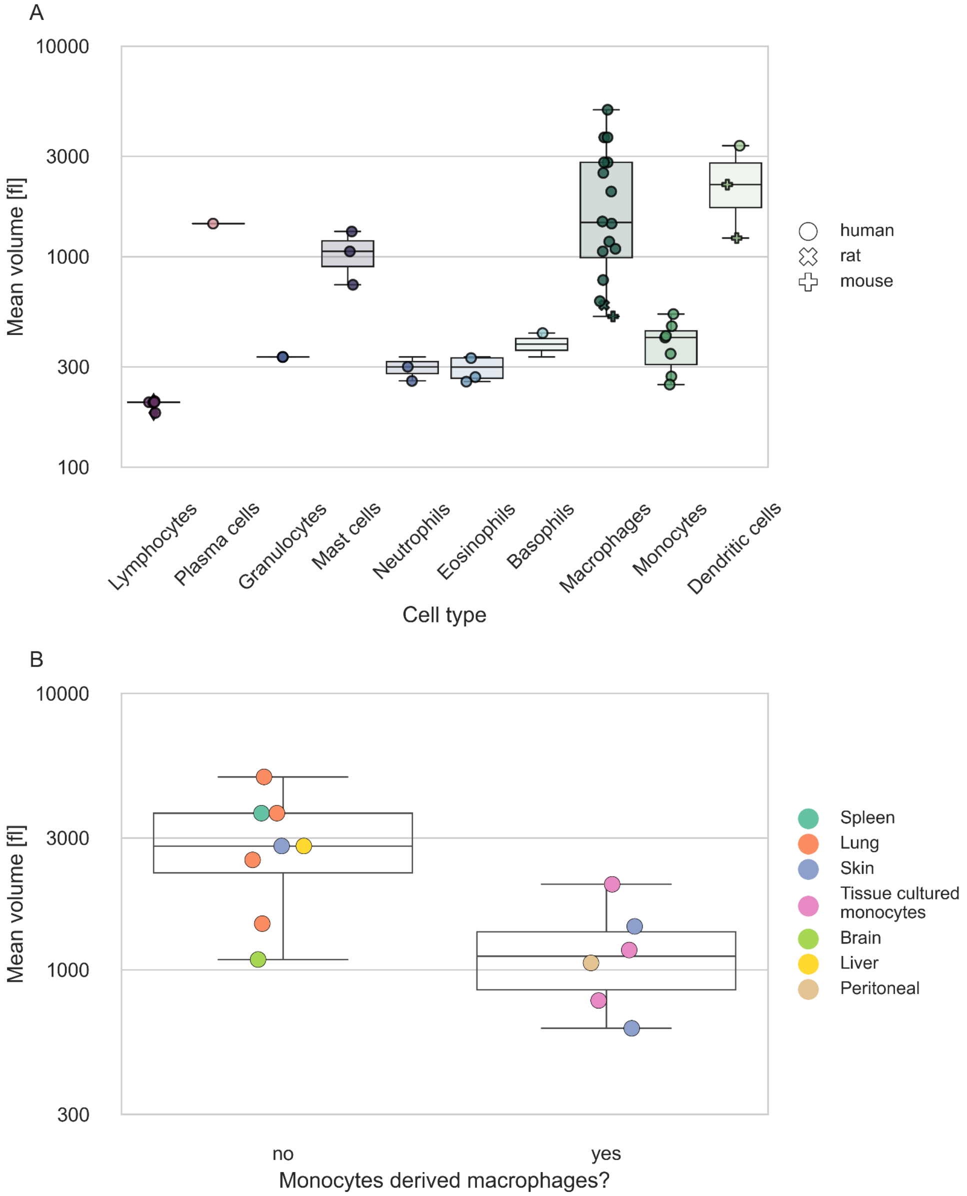

Fig. S6: Cellular volume of immune cells based on the literature. *Estimates for the volume of each cell type were gathered from the literature (see Method section).* ***A.*** *Volume estimates for each cell type are presented via a scatter and boxplot. The box represents the interquartile range (percentiles 25-75), and the whiskers represent the maximal range of the distribution apart from outliers (defined as data points exceeding the interquartile range by a factor of 1.5). Most values were derived from human-based data. The few cases in which rodent data were considered are marked by specific shaped markers. y-axis is given in log scale* ***B.*** *Macrophages exhibited a wide range of sizes that appeared to be influenced by the tissue they reside in (16, 21, 22, 26). We collected estimates from various tissues and categorized them into two groups: tissues*

with continuous replenishment from monocytes and those without (27). The y-axis is given on a log scale.

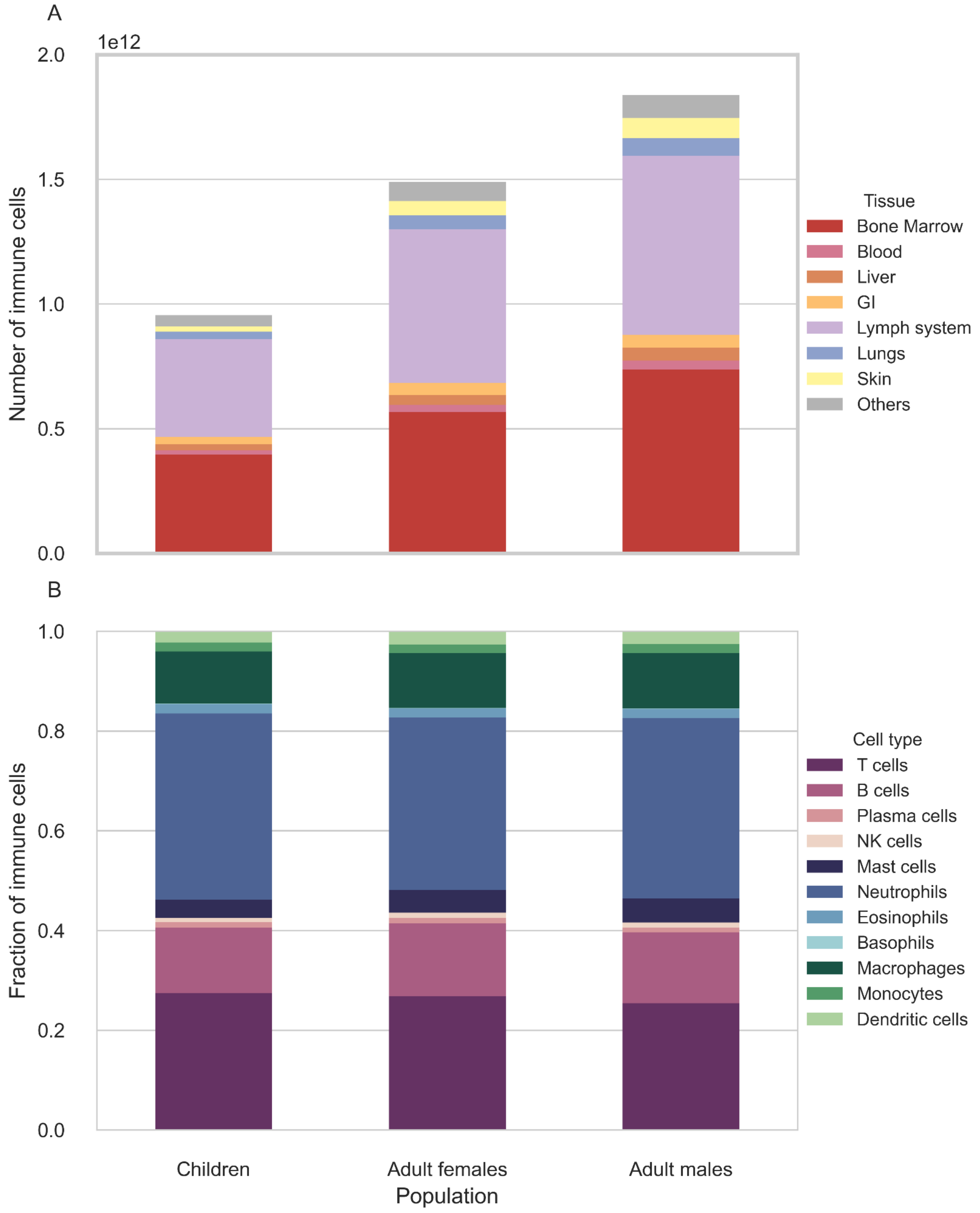

Fig. S7: Effects of sex and age on the immune cell distribution. *The distribution of immune cells in a reference adult man (weighing 73 kg) is compared to that of a reference 10-year-old child (32 kg) and a reference adult woman (60 kg). The number of immune cells was calculated by multiplying tissue-specific immune cell densities by the*

mass of the tissues. Immune cell densities used for children, women, and men were averaged densities pooled without regard to sex and age due to a lack of data. ***A.*** *The total number of immune cells by tissue.* ***B.*** *The distribution of immune cells by cell types.*

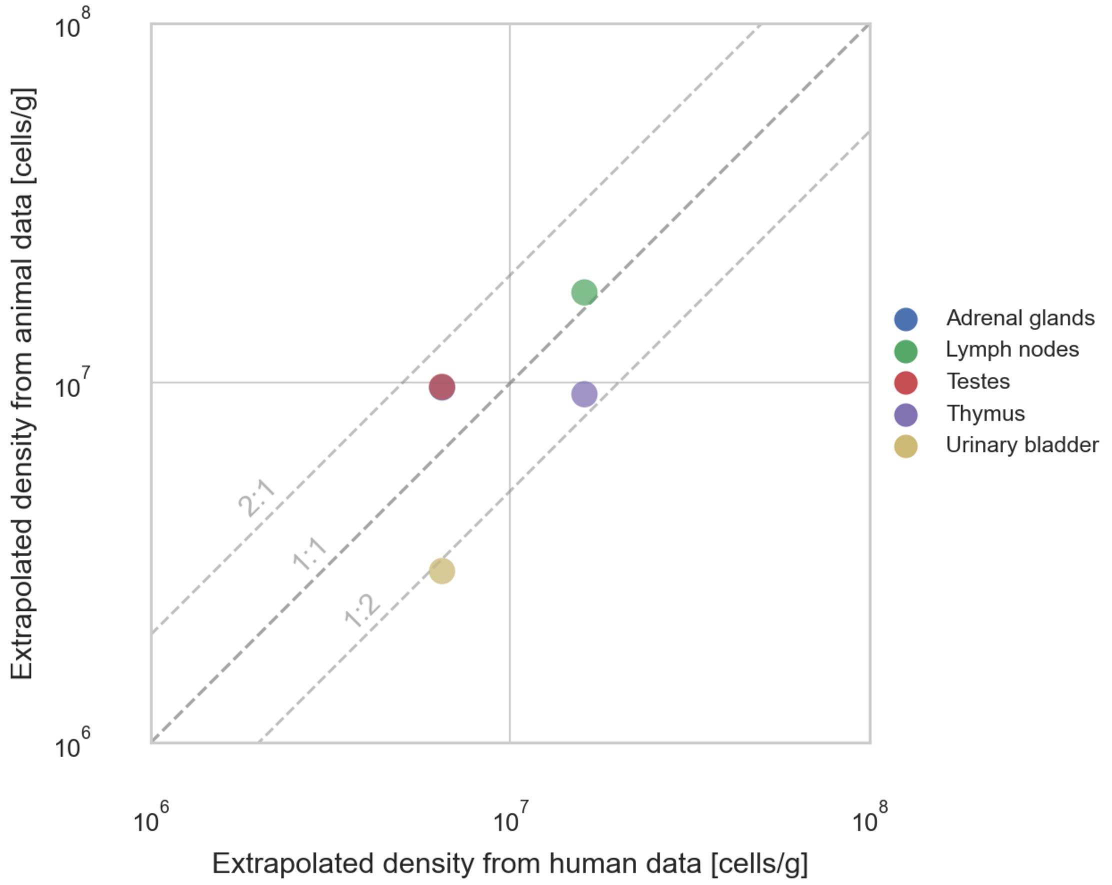

***Fig. S8: Comparison of extrapolation of macrophage density from animal data compared to extrapolation from similar tissues in humans**. Extrapolation from similar tissues was done via the geometric mean of the tissue group, as defined in the Methods section. The diagonal lines represent constant ratios between the estimates. Values are within two-fold differences.*

Dataset S1 (separate file). A dataset of immune cell densities and cellular mass was extracted from the literature for the calculation of the immune cell distribution. The data is analyzed by Python notebooks to derive the final estimates for immune cell distribution.

Dataset S2 (separate file). The final estimates of immune cell distribution in the human body. The estimates are given in terms of number and mass from various perspectives: aggregations by tissues or cell types.
